# Exploring a Therapeutic Gold Mine: The Antifungal Potential of the Gold-Based Antirheumatic Drug Auranofin

**DOI:** 10.3390/ijms26167909

**Published:** 2025-08-16

**Authors:** Jingyi Ma, Wendy van de Sande, Bernhard Biersack

**Affiliations:** 1 Department of Medical Microbiology and Infectious Diseases, Erasmus MC, University Medical Center Rotterdam, Dr. Molewaterplein 40, 3015 GD Rotterdam, The Netherlands; m.jingyi@erasmusmc.nl (J.M.); w.vandesande@erasmus.nl (W.v.d.S.); 2 Organic Chemistry Laboratory, University of Bayreuth, Universitätsstrasse 30, 95440 Bayreuth, Germany

**Keywords:** antifungal drugs, metal-based drugs, auranofin, gold complexes, mycoses

## Abstract

Human fungal infections comprise systemic mycoses as well as various skin diseases. Rising case numbers along with inefficient therapies and the appearance of drug-resistant strains unleashed a considerable health problem over the last years. Thus, the identification and development of new antifungal drugs is mandatory, which can include the design of new antifungals, or, more time saving, the repurposing of known drugs already applied for the therapy of other human diseases. The orally applicable gold-based drug auranofin has been used for the treatment of rheumatoid arthritis since the 1980s. However, auranofin also showed marked activity against various cancers, microbes, parasites, and viruses. Facing a pressing need to find new drug candidates against mycoses, especially against those listed in the WHO fungal pathogen priority list, we have summarized the eminent antifungal activities of auranofin in this review. Given its established safety profile and broad-spectrum activity, auranofin represents a promising candidate for repurposing in antifungal therapy. The mechanism of action of auranofin was correlated with thioredoxin reductase inhibition, but other modes of action such as interference with mitochondrial protein import and NADH kinase were also described and discussed. A selection of promising antifungal gold complexes was also provided. Pertinent literature is covered until 2025.

## 1. Introduction

In the past decades, the number of patients at risk for developing a mycosis has risen considerably [1,2,3]. It was estimated that human mycoses attributed to ca. 3.8 million deaths in 2023 [4]. Despite the increase in fungal infections over the years, the number of antifungal agents able to treat these infections has remained low. At the moment, there are only a few classes of antifungal agents available to treat fungal infections. These include antimetabolites (flucytosine), suppressors of ergosterol biosynthesis/function (the natural polyene amphotericin B and various synthetic azole drugs such as fluconazole, itraconazole, voriconazole, and posaconazole), 1,3-β-glucan synthase inhibitors (e.g., cyclic lipopeptides of the echinocandin class such as caspofungin and micafungin, the triterpenoid ibrexafungerp), and the pyridine-isoxazole-based glycosylphosphatidylinositol biosynthesis inhibitor fosmanogepix [5]. Furthermore, resistance to azoles and echinocandins is on the rise and was widely reported in *Candida* and *Aspergillus* species. The situation becomes even more problematic with the emergence of inherently drug-resistant species such as *Candida auris* and otherwise insensitive fungal strains (e.g., eumycetoma fungi with a field treatment efficacy of only 25–35%) [6,7,8]. Only a few drugs belonging to new classes such as olorofim are currently under development. Thus, the design of new antifungal treatment options is urgent. Such efforts can include an optimized application of already existing antifungal drugs, the design of new drugs, and the repurposing of drugs with antifungal potential that are applied for other human ailments [9]. Addressing new fungal targets to overcome resistance mechanisms is prioritized, and new promising targets include fungal Hsp90 (heat-shock protein 90), HDACs (histone deacetylases), protein kinases, and DHODH (dihydroorotate dehydrogenase), for example [10,11,12,13]. However, the development of new safe drugs is expensive and time-consuming. Thus, the repurposing of already existing drugs can accelerate the establishment of new antifungals in clinical practice [14]. In particular, metal-based drugs can fulfill the urgent need to develop new and promising antifungals [15].

Gold has fascinated humans since the beginning of time. Medicinal properties were attributed to gold by various ancient cultures, and this knowledge has reached modern times via traditional Indian (Ayurveda) and Chinese medicine (TCM) [16,17,18]. In addition to elementary gold, the discovery of alchemists to dissolve gold with specific oxidative acidic solutions (e.g., aqua regia) has expanded the medical application of gold by compounds such as salts [19,20,21]. Until today, the dissolution of gold is an active and important field of chemical research [22,23]. The first antimicrobial activities of gold were described in the 19th century when André-Jean Chrestien (1811) and Robert Koch (1890) identified the antibacterial and tuberculostatic activities of gold salts such as gold sodium chloride and gold cyanide K[Au(CN)_2_], respectively [24]. The Danish physiologist Holger Møllgaard introduced sanocrysin (sodium aurothiosulfate) for the therapy of tuberculosis in 1925, which was frequently used in Europe until 1935 despite its inefficacy and toxic side-effects [25]. Because Jacques Forrestier erroneously considered rheumatoid arthritis (RA) as an infectious disease in analogy to tuberculosis, he began to study the effects of gold drugs on RA and disclosed the first report of rheumatoid patients successfully treated with a gold compound, gold thiopropanol sodium sulphonate (allochrysine), in 1929 [26,27]. Meanwhile, scientific research on biologically active and medically relevant gold compounds and complexes has become more and more important [28,29,30]. Forrestier’s discovery eventually led to the development of the second-generation gold drug auranofin (Ridaura^TM^), which was identified as an anti-arthritic drug in 1972 and approved by the FDA for the therapy of RA 40 years ago, in 1985 [31,32].

Auranofin is a linear gold(I) complex with tetra-*O*-acetylthioglucoside and triethylphosphine ligands. In contrast to other injectable polymeric gold-based drugs (sodium aurothiomalate/myochrysine, aurothioglucose/solganal) used for RA chrysotherapy, auranofin is orally applicable. Meanwhile, it was reconsidered for the treatment of various other human ailments [32,33,34]. These include several forms of cancer and various microbial, viral, and parasitic infections [35,36,37,38,39,40,41]. There is also growing evidence that auranofin is a potent antifungal agent.

Auranofin can become a suitable drug for the therapy of human mycoses for various reasons. As mentioned above, auranofin has already shown promising anti-infective activities (antiviral, antibacterial, and antiparasitic activities). Its chemical and pharmacological properties are well-known, and its suitability as a human drug is well-established after 40 years of clinical application as an oral RA therapy (see Section 3.1 and Section 3.2). Auranofin can be useful for the treatment of resistant/insensitive fungal strains and can lead to considerable synergy effects in combination therapies (see Section 4.1 and Section 4.2). Its mechanisms of action (e.g., inhibition of thioredoxin reductase/TrxR, of vital mitochondrial proteins, etc.) add well to those of the existing classes of fungicides (see Section 5.1). Furthermore, the simple synthesis and chemical structure of auranofin can be a blueprint for the development of new gold complexes with sound antifungal activities (see Section 5.2). Thus, auranofin stands out among other drugs considered for repurposing such as niclosamide, ebselen, or disulfiram. Niclosamide is clinically applied only for the therapy of intestinal worm infections and has a low oral bioavailability, requiring formulation strategies to achieve an application for the therapy of systemic diseases [42]. Although in clinical trials, the TrxR inhibitor ebselen has not been approved as a human drug yet, and thus the development of ebselen as an antifungal drug has to pass several hurdles [43]. The alcohol dehydrogenase inhibitor disulfiram, applied for the therapy of alcohol abuse, displayed considerable anti-mycotic properties but has severe side-effects [44]. Moreover, its interactions with metal ions and the promising antifungal activity of disulfiram in combination with copper ions underscore the potential of the development of metal-based antifungals such as auranofin [45].

## 2. Methodology

PubMed, SciFinder, and Google were used for a literature search. In addition, PubChem was applied for literature search using the IUPAC names of auranofin and other antifungal gold compounds. Only papers written in English language from well-established and recognized scientific journals were cited. Contributions from English and German textbooks about metal-based drugs, bioinorganic chemistry, and related fields were also considered. Moreover, relevant content of two active websites (Drugs for Neglected Diseases initiative/DNDi and Mycetoma Open Source/MycetOS) was mentioned.

For the introduction, the content of pertinent reviews using the keywords “mycoses”, “antifungal drugs”, “auranofin”, “cancer”, and “infectious diseases” was cited, and only papers written by experts in the described fields were considered. For the results section, pertinent literature on the chemistry and pharmacology of auranofin was collected (using the keywords “auranofin” and “pharmacology”) and cited, followed by a PubMed literature search for the antifungal properties of auranofin (keywords “auranofin” and “antifungal”). Antifungal properties of auranofin were listed and ranked according to the fungal pathogen priority recommended by the World Health Organization (WHO). Crucial mechanisms of auranofin were selected for a deeper discussion and a possible outlook (using the keywords “auranofin”, “thioredoxin reductase”, “reactive oxygen species”, “antifungal honokiol”, “mitochondrial targets”, “Mia40” and “Pos5 NADH kinase”, “NF-κB inhibitor” and “velvet proteins”, “pentamidine”, “membrane disruption”, and “gold uptake”). The described antifungal gold complexes were selected based on their anti-mycotic relevance (keywords “gold complexes” and “antifungal”), while studies using gold nanomaterials were not considered.

The literature survey was carried out between March and July 2025. Pertinent literature published until July 2025 was covered in this review.

## 3. Auranofin: Chemistry and Pharmacology

A thorough understanding of the chemistry and pharmacology of a drug candidate is required to evaluate its drug-like potential. In particular, metal complexes can possess properties that differ distinctly from purely organic drug molecules. Since the gold(I) complex auranofin is an approved RA drug, there is much data available in terms of its chemical and pharmacological properties. Thus, relevant remarks on the chemistry and pharmacology of auranofin are given below, which might be useful for its repurposing as an antimicrobial agent.

### 3.1. Notes on Auranofin Chemistry

**Scheme 1:**
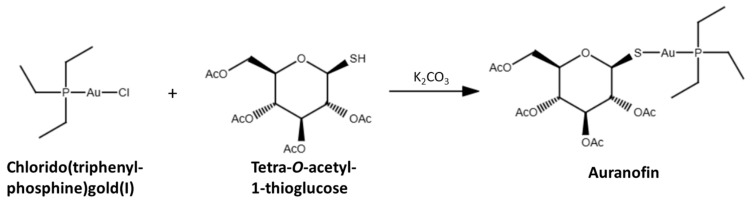
Synthesis of auranofin from chlorido(triphenylphosphine)gold(I) and 2,3,4,6-tetra-*O*-acetyl-1-thio-β-D-glucose under basic conditions in the presence of K_2_CO_3_.

Auranofin, triethylphosphine(2,3,4,6-tetra-*O*-acetyl-β-1-D-thiopyranosato-*S*)gold(I) (C_20_H_34_AuO_9_PS, MW 678.5 g/mol, CAS No. 34031-32-8), is a linear neutral gold(I) complex coordinated to triethylphosphine and 2,3,4,6-tetraacetyl-1-thio-β-D-glucose ligands. It is a colorless solid and optically active due to the carbohydrate ligand. Auranofin crystallizes from ethanol as a single polymorph (melting point of 114.74 °C, 387.9 K). Its X-ray structure was solved and displayed an S-Au-P angle of 172.73°, confirming the linear character of this gold(I) complex. Another crystal polymorph reported in older literature could not be reproduced [46]. While salts of Au(I) are usually unstable in aqueous media leading to a disproportionation to metallic gold and Au(III), the “soft” phosphor and sulfur ligands stabilize the oxidation state of the Au(I) center, which is a “soft” acid, in gold(I) complexes such as auranofin [24]. The synthesis of auranofin is straightforward, and the drug can be easily prepared from the reaction of chlorido(triethylphosphine)gold(I) with 2,3,4,6-tetra-*O*-acetyl-1-thio-β-D-glucose under basic conditions (K_2_CO_3_, CH_2_Cl_2_/H_2_O, 0 °C to room temperature) (Scheme 1) [47]. Alternatively, *S*-(2,3,4,6-tetra-*O*-acetylglucopyranosyl)pseudothiourea hydrobromide can be used instead of 2,3,4,6-tetra-*O*-acetyl-1-thio-β-D-glucose for this reaction [48].

### 3.2. Pharmacology of Auranofin

The auranofin precursor chlorido(triethylphosphine)gold(I) also showed anti-arthritic properties, but it exhibited considerable adverse effects as an oral drug [49]. Thus, for RA therapy, a more tolerable orally applicable gold drug was required and developed in form of the prodrug auranofin upon replacing the chlorido ligand by an *O*-acetylated thioglucose ligand. The triethylphosphine ligand of auranofin contributes to the lipophilic character of the gold(I) complex while the thiosugar ligand is labile and readily released, leading to drug activation followed by reaction with nucleophilic targets, in particular, with sulfur- and selenium-containing proteins and peptides [50,51]. The Au-P bond is more stable, but the phosphine ligand also undergoes dissociation and oxidation by the time, leading to the triethylphosphine oxide (Et_3_P=O) metabolite. A particularly rapid Et_3_P=O formation was identified for the seleno-auranofin derivative upon reaction with serum albumin [52]. The metal-free ligands of auranofin have no significant biological properties, while in several cases the (triethylphosphine)gold(I) fragment is required for activity [53,54]. Important biologically active metabolites of orally administered auranofin are chlorido(triethylphosphine)gold(I) in the stomach and aurocyanide/dicyanoaurate(I) [Au(CN)_2_]^−^ upon longer incubation times by reaction with cyanide ions naturally occurring in the blood [55,56]. The discovery that the linear aurocyanide complex is a bioactive metabolite of gold anti-rheumatics closes the arch to Robert Koch’s findings of its activity against tuberculosis from the late 19th century.

Early studies on the immunomodulatory mechanisms of auranofin revealed an inhibition of lysosomal enzyme release from cells, a suppression of antibody production in arthritic rats, and an inhibition of antibody-dependent complement lysis [54]. Further immune-suppressive effects of auranofin include suppression of lymphoblastogenesis and lymphocyte function [57]. Conversely, auranofin stimulated IL-6 and IL-8 secretion in monocytes, activated T cell immunity associated with interferon-γ upregulation, and enhanced the cytotoxic activities of natural killer (NK) cells analogously to interferon but by a different mechanism. However, NK activation was only observed at low doses of auranofin, while high doses inhibited NK activity [58]. The anti-inflammatory activities of auranofin are based on the suppression of pro-inflammatory factors such as COX-2 (cyclooxygenase-2), NOS (nitric oxide synthase), NF-κB (nuclear factor-κB), and TrxR, as well as on the activation of peroxyredoxin-1 and Nrf2 (nuclear factor erythroid 2-related factor 2) [19]. The coordination of auranofin to crucial (seleno-)cysteine residues of IκB kinase (IKK), leading to NF-κB inactivation, and of reduced TrxR was documented [59,60]. It is possible that auranofin can suppress the notorious and life-threatening “cytokine storm” that occurs during severe infections and associated hyperinflammation processes [37]. But since various human mycoses preferably develop in immunocompromised people, deeper investigations are required to evaluate the role of the immunomodulatory activities of auranofin in fungal infections.

Auranofin is an orally applicable drug, and plasma concentrations upon ingestion reached values of 25% of the administered dose in humans with peak values of 6–9 µg/100 mL after 1–2 h, and plasma half-lives of 15–25 days [24]. A considerable amount of auranofin reacts with albumin by ligand exchange and coordination to albumin cysteine [61]. Auranofin is a slowly acting RA drug, and long-term medication (e.g., for one year) as well as patient monitoring are required for RA therapy to achieve a sustained therapeutic effect and to manage side-effects [62]. The recommended oral dose of auranofin for RA patients is 3 mg twice daily (=6 mg per day) followed by maintenance therapy with 3 mg once/twice daily, and the most common adverse effect of auranofin is diarrhea (especially at higher doses), followed by rash, stomatitis, conjunctivitis, and proteinuria, while thrombocytopenia and bone marrow suppression were only rarely observed [24].

To avoid frequently occurring diarrhea, oral doses of 3–6 mg per day, or below, should also be considered when repurposing auranofin for the treatment of other human diseases. More recent clinical studies of auranofin underline the low toxicity of this drug and its efficacy against glioblastoma in combination with nine other drugs (CUSP9v3, adverse effects: reduced lymphocyte numbers, increased alanine aminotransferase level, and nausea) and HIV-1 in combination with intensified antiretroviral therapy/ART (ART + dolutegravir + maraviroc, adverse effect: reduced CD4^+^ T-cell numbers) [63,64]. A phase 1 trial of auranofin (6 mg/day orally for 7 days followed by adverse effect monitoring until day 126) in healthy volunteers in preparation for a clinical trial with patients suffering from parasitic intestinal infections (*Entamoeba histolytica*, *Giardia lamblia*) revealed mild adverse effects (gastrointestinal problems and headache), maximum plasma concentrations of 0.312 µg/mL after 7 days, half-life of 35 days, and high drug concentrations of 13 µM in the feces after 7 days, which was much higher than required for effective doses against *E. histolytica* (25 × IC_50_) and *G. lamblia* (4 × IC_50_) [65]. In a phase 2 trial with pulmonary tuberculosis patients, auranofin (3 mg/day for 7 days, then 6 mg/day until day 112) combined with standard therapy showed no therapeutic advantages in comparison with standard therapy alone. Moreover, two patients showed life-threatening adverse effects (thrombocytopenia and intra-abdominal sepsis, respectively). The appearance of thrombocytopenia in one of these patients was correlated with auranofin and was resolved after discontinuation of auranofin treatment. It could not be excluded that the intra-abdominal sepsis in the second patient was also caused by auranofin, and thus, it was classified as a suspected unexpected serious adverse reaction, also because of previous reports of severe colitis in RA patients undergoing auranofin therapy [66]. The fact that the tuberculosis patient with sepsis died underscores the necessity of close monitoring of persons receiving auranofin therapy.

In comparison with the adverse effects of currently applied antifungal drugs, auranofin shows a promising toxicity profile. Azoles also lead to gastrointestinal problems (nausea, vomiting, abdominal pain, diarrhea), but show additional hepatotoxic and cardiotoxic effects, which are rare or absent in auranofin-based therapies. Flucytosin therapy is accompanied by liver toxicity and hematological side-effects (leukopenia, thrombocytopenia). In particular, polyene drugs are known for their high toxicity. In addition to gastrointestinal and hematological adverse reactions, amphotericin B, also known as “amphoterrible” by clinicians, has a high nephrotoxic potential, which could be reduced by suitable formulations such as liposomal amphotericin B [67].

## 4. Antifungal Activities of Auranofin

The published activities of auranofin against human mycoses are summarized below. The activities were described following the WHO fungal priority pathogens list (critical-priority, high-priority, and medium-priority groups), which was established to prioritize research efforts and to accelerate medicinal outcomes [68]. Moreover, sound activities of auranofin against fungal pathogens not listed by the WHO were also outlined. The potential of combinations with other antifungal drugs was also outlined, and the structures of combination partners leading to synergy and/or additive effects in fungal pathogens were provided in the Supplementary Materials File (Figure S1).

### 4.1. Antifungal Activities of Auranofin–WHO Critical- and High-Priority Group Pathogens

The species *Candida auris*, *Candida albicans*, *Cryptococcus neoformans*, and *Aspergillus fumigatus* were classified as critical-priority fungal pathogens. Three further *Candida* species (*Candida glabrata*, *Candida tropicalis*, and *Candida parapsilosis*), as well as *Fusarium* species and the causative agents of eumycetoma and histoplasmosis, were grouped as high-priority fungal pathogens [68]. Candidiasis, aspergillosis, and cryptococcosal meningitis pose severe clinical problems with a global impact and a high demand for potent and safe antifungal drugs. Antimicrobial studies using various gold complexes including auranofin revealed broad-spectrum activities and multiple mechanisms of action, suggesting a high probability of efficacy even in drug-resistant strains, as well as synergy effects in combination with other drugs. Accordingly, a reduced risk of resistance formation can be expected upon chrysotherapy. In addition, the modification of ligand molecules can quickly lead to optimized gold complexes (see Section 5.2) [38,69].

**Figure 1:**
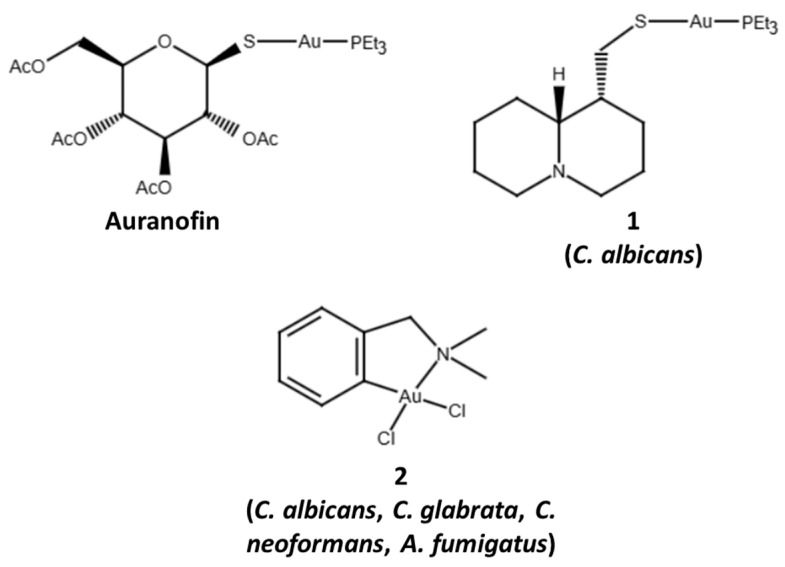
Structures of auranofin and two early described antifungal gold complexes **1** and **2** (sensitive critical- and high-priority group fungi in brackets).

Already in 1999, two reports about antifungal gold complexes with activities against now critical- and high-priority pathogens were disclosed (Figure 1). A (triethylphosphine)gold(I) complex **1** with the quinolizidine-based sulfur ligand lupinylsulfide (derived from the natural alkaloid lupinine) instead of the thiosugar of auranofin exhibited antifungal activity against *C. albicans* (MIC/minimal inhibitory concentration = 7.81 µg/mL) [70]. A dichloridogold(III) complex **2** with a damp (dimethylaminomethylphenyl) chelating ligand was active against a panel of pathogenic yeast and mold strains (MIC = 2.5–25 µg/mL, *C. albicans*, *C. glabrata*, *C. neoformans*, and *A. fumigatus*) [71]. More gold(I) and gold(III) complexes with antifungal activities are described below (see Section 5.2). In the coming passages, the antimycotic potential of auranofin in critical- and high-priority fungal pathogens is outlined in detail.

The screening of drug libraries was decisive for the identification of the antifungal activities of auranofin. First evidence of a considerable antibiofilm activity of auranofin against *C. albicans* fungi was reported by Lopez-Ribot and Ramasubramanian in 2013. Screening of the Prestwick Library (Prestwick Chemical, France) of 1200 FDA-approved off-patent small-molecule compounds for inhibition of *C. albicans* SC5314 biofilm formation at drug doses of 20 µM identified auranofin as an active compound (94% inhibition of biofilm formation). Auranofin was slightly more inhibitory in preformed biofilms than in newly forming biofilms, and activities were close to the steady-state mean gold blood concentration (ca. 3.5 µM) in patients undergoing auranofin RA therapy (Table 1). In terms of preformed *C. albicans* biofilm destruction, auranofin was more potent than the polyene antifungals amphotericin B and nystatin, while all tested azoles and other antifungal drugs (butenafine, ciclopirox, flucytosine) were inactive against preformed biofilms [72]. The pronounced antibiofilm properties of auranofin were also detected in dual biofilms of *Candida* species and bacterial pathogens such as *Staphylococcus aureus*. Mixed biofilms of pathogenic bacteria and fungi are especially resistant to antibiotics. But auranofin exhibited strong inhibition of dual *S. aureus*/*C. albicans* biofilms, as well as synergy effects in combination with sub-MIC doses of amphotericin B in dual *S. aureus*/*C. albicans* and *S. aureus*/*C. parapsilosis* biofilms. Notably, the presence of fetal bovine serum reduced the antibiofilm properties of auranofin, possibly by interaction with serum components [73]. Polyurethane/PU-catheters coated with auranofin also strongly suppressed *C. albicans* biofilms as well as dual microbe (*S. aureus*/*C. albicans*) biofilms in comparison to uncoated catheters, and the antibiofilm activity of catheters coated with 10 mg/mL auranofin was proven in a murine subcutaneous model [74]. Thus, auranofin has the potential to prevent catheter-mediated bloodstream infections.

Another screening of compound libraries (Enzo FDA-approved drug library and FIMM oncology collection) from 2014 revealed antifungal activities of auranofin against *C. albicans* (SC5314 and the clinical UBC3-7922 strain). However, two *C. glabrata* strains (including the ATCC 90030 strain) were less sensitive to auranofin, which is in accordance with a lower susceptibility of *C. glabrata* fungi to fluconazole treatment (Table 1) [75]. Notably, in a different study from 2016, auranofin was active against *C. glabrata* ATCC 90030 and a majority of *C. glabrata* clinical isolates; only two isolates were less susceptible. In addition, auranofin was active against four *C. tropicalis* strains [76].

The research group that identified the biofilm-disruptive properties of auranofin in *C. albicans* in 2013 (Lopez-Ribot and Ramasubramanian, University of Texas, San Antonio) also investigated the antifungal activities of auranofin against a broad panel of clinically relevant yeasts including *Candida* spp. four years later. Again, all 13 *C. albicans* clinical isolates used in this study were sensitive to auranofin treatment with MIC values of 0.25–1 µg/mL including four isolates resistant to fluconazole. The *C. glabrata* isolates were less susceptible to auranofin, and so were the *C. parapsilosis* isolates, which were sensitive to fluconazole [77]. Another study from the same year disclosed the antifungal activity of auranofin against various *Candida* species (*C. albicans*, *C. glabrata*, *C. tropicalis*, and *C. parapsilosis*) as well as biofilm formation inhibition in the *C. albicans* ATCC 10231 strain at 16 µg/mL (8 x MIC) in contrast to flucytosine and fluconazole (inactive against biofilms). *Saccharomyces cerevisiae* wildtype and deletion strains were used to identify Mia40 (mitochondrial IMS import and assembly pathway 40 kDa) as a possible mitochondrial target for auranofin in yeasts. Auranofin blocked the interaction of Mia40 with the substrate Cmc1, thus preventing mitochondrial import of Cmc1 (Table 1) [78]. Mia40 is involved in the Mia40-Erv1 (essential for respiratory growth and viability 1) mitochondrial protein import pathway by formation of disulfide bridges with suitable substrates (disulfide relay system, in cooperation with the sulfhydryl oxidase Erv1) that enter the mitochondrial intermembrane space via Tom (translocase of the outer membrane) channels [79]. Mia40 is not the sole target in fungal mitochondria, and the NADH kinase Pos5 (peroxide sensitive 5) was identified as another mitochondrial target of auranofin (see Section 4.2) [80].

To enhance the antifungal activity of auranofin against *C. albicans*, the combination with another promising compound, the antiparasitic and antifungal drug pentamidine, was evaluated. Indeed, synergy effects were observed in all five *C. albicans* strains used in this study, which included the sensitive ATCC 64548 strain, the fluconazole-resistant ATCC 64550 strain, and three multidrug-resistant clinical isolates (Table 1). The MIC values of auranofin and of pentamidine were reduced upon combination of both drugs. Pentamidine increased the membrane permeability of fungal cells, leading to a higher cellular uptake of auranofin (twice as high as in auranofin monotherapy without pentamidine). Moreover, the combination of both drugs was hemocompatible at effective concentrations [81]. In a recently disclosed study, auranofin inhibited biofilms of multidrug-resistant *C. auris*, showed synergy effects in combination with pentamidine, and increased survival rates in a *Tenebrio molitor* larvae-based *C. auris* infection model as a single agent and at lower doses together with pentamidine. Mechanistically, pentamidine treatment led to leaky *C. auris* membranes associated with enhanced protein and nucleotide extravasation, and caused mitochondrial damage (Table 1). These effects were achieved at distinctly lower pentamidine concentrations when combined with auranofin [82].

*C. neoformans* is another pathogenic yeast that causes severe health problems in humans. Thus, in addition to *Candida* yeasts, auranofin was often also evaluated in *Cryptococcus* species. Auranofin revealed a remarkable potency in 34 *C. neoformans* clinical isolates, which indicates a broad spectrum antifungal activity of auranofin. However, *C. neoformans* biofilms were not inhibited by auranofin, which is in stark contrast to its antibiofilm properties in *C. albicans*. Additive effects were observed in combination with pro-oxidant diamide and H_2_O_2,_ while glutathione reduced the efficacy of auranofin in *C. neoformans,* which is in line with a proposed targeting of the vital cryptococcal thioredoxin system by the gold(I) drug. Additive effects on *C. neoformans* were also found in combination with fluconazole or amphotericin B (Table 1) [76]. Moreover, auranofin showed comparable activities to fluconazole against three *C. neoformans* isolates (Table 1) [77]. In an in vivo study *Caenorhabitis elegans* worms infected with *C. neoformans* isolate NR-41292 (isolated from the cerebrospinal fluid of a Chinese patient in 2012), auranofin (8 µg/mL) distinctly reduced the mean fungal load and outperformed the approved antifungals fluconazole and flucytosine (both 8 µg/mL) [78]. The anti-cryptococcal activities of auranofin were confirmed in a different study using four *C. neoformans* strains (Table 1) [83].

Auranofin also showed activity against clinical isolates of the pathogenic mold *A. fumigatus* (Table 1) [77]. A more recent study revealed antifungal and fungicidal activities of this gold drug against various *Aspergillus* molds including itraconazole-resistant *A. fumigatus*. Auranofin exhibited antibiofilm activity in itraconazole-resistant *A. fumigatus* by suppression of SomA and MedA. Moreover, synergy effects were observed in combination with amphotericin B or itraconazole in the resistant strain. Notably, auranofin coordinated to the *Af*TrxR cysteines C145 and C148, leading to inhibition of this vital fungal enzyme, while the gold complex also downregulated *Af*TrxR transcription (Table 1) [84].

Next to the critical-priority fungal pathogens, auranofin also had activity against high-priority fungal pathogens. So far, there are 69 fungal species listed as causative agents of eumycetoma, also known as. “Madura foot”. Together they are listed as high-priority fungal pathogens. The most common eumycetoma causative agents are *Madurella mycetomatis*, *Falciformispora senegalensis*, *Trematosphaeria grisea*, *Scedosporium boydii*, and *Medicopsis romeroi* [85,86]. It was demonstrated that auranofin (from the Pathogen Box drug library of the Medicines for Malaria Venture/MMV) inhibits the growth of *M. mycetomatis* with an IC_50_ of 17.1 µM and an IC_90_ of 45.8 µM. Notably, testing of commercially available auranofin powder led to higher activities (IC_50_ = 11.2 µM, IC_90_ = 15.0 µM, Table 1) [87]. The Pathogen Box samples were delivered as DMSO stock solutions and were probably exposed to DMSO for longer times than the freshly prepared stocks from the compound powder, which might explain the observed activity discrepancies.

The efficacy of auranofin against various drug-resistant *Scedosporium* species and *Lomentospora prolificans* (formerly known as *Scedosporium prolificans*), which can manifest as systemic infections and as localized skin infections (eumycetoma), is described in Table 2 (see Section 4.2) [88,89,90].

Although not officially listed by the WHO as a fungal NTD (neglected tropical disease), the endemic mycosis histoplasmosis is also considered as a neglected disease and categorized as a high-priority fungal infection [68]. *Histoplasma capsulatum* is the causative agent of histoplasmosis, leading to casual outbreaks and chronic pulmonary disease in immunocompetent people, as well as to lethal infections in immunocompromised patients such as HIV-infected persons in Latin America, especially in Brazil [91,92]. In North America, *H. capsulatum* is endemic in the vast regions of the valleys of the Ohio and Mississippi rivers [3]. Auranofin inhibited the growth of 15 *H. capsulatum* strains and exhibited fungicidal activities in eight of these strains. Moreover, auranofin was active against intramacrophageal *H. capsulatum*, and the combination of auranofin plus amphotericin B was synergistic in five *H. capsulatum* strains. Auranofin reduced the cell size of *H. capsulatum* G217B and modulated the expression of various virulence genes (suppression of Hsp70 and YPS3, induction of thioredoxin reductase). In vivo, auranofin (5.7 mg/kg) treatment of *T. molitor* larvae infected with *H. capsulatum* G217B led to increased survival rates (similar to 5.7 µg/kg itraconazole) in comparison to untreated infected larvae (Table 1) [93].

**Table 1:** Activity of auranofin against critical- and high-priority fungal pathogens.

Pathogen(s) (Priority)	Activity			Mechanism(s)	Ref.
	In Vitro	In Vivo	Combinations		
*C. albicans* (critical)	Antibiofilm (IC_50_ = 5.1 µM for preformed, 6.1 µM for newly forming biofilms), more active than AmB ^1^ (IC_50_ = 7.5 µM) and nystatin (IC_50_ = 8.9 µM)	-	-	-	[72]
*C. albicans* (critical)	Antibiofilm against mixed biofilms (*S. aureus*/*C. albicans*) on PU catheters	Antibiofilm against mixed biofilms on auranofin-coated catheter (10 mg/mL) in a murine subcutaneous model	-	-	[74]
*C. albicans* (critical)	Antifungal (MIC = 0.061–0.68 µg/mL, MIC_0.3_ = 0.07–0.08 µg/mL)	*-*	*-*	*-*	[75]
*C. albicans* (critical)	Antifungal (MIC = 0.25–1 µg/mL), active against fluconazole-resistant strain	*-*	*-*	*-*	[77]
*C. albicans* (critical)	Antifungal (MIC = 3.9–15.6 µg/mL, MIC = 125 µg/mL in a resistant strain)	*-*	Synergy with pentamidine (MIC = 0.9–2.0 µg/mL for auranofin), sensitization of a resistant strain (MIC = 31.3 µg/mL for auranofin), hemocompatible	Increased gold uptake, leaky cell membranes by pentamidine	[81]
*C. auris* (critical)	Fungistatic, antibiofilm (128 µg/mL)	Increased survival in infected *T. molitor* (128 µg/mL as single agent, 32 µg/mL in combination with 8 µg/mL pentamidine)	Synergy with pentamidine	Leaky fungal membranes by pentamidine, mitochondrial damage	[82]
*Candida* spp. (critical/high)	Antifungal (MIC ~ 2 µg/mL for *C. albicans*, 0.25–1 µg/mL for *C. glabrata*, 0.125–0.5 µg/mL for *C. tropicalis*)	-	-	-	[76]
*Candida* spp. (critical/high)	Antifungal (MIC = 1–16 µg/mL for *C. albicans*, 8 µg/mL for *C. glabrata*, 4–16 µg/mL for *C. tropicalis*, 4 µg/mL for *C. parapsilosis*), antibiofilm (*C. albicans*)	-	-	Mia40 inhibition, interference with mitochondrial protein import ^3^	[78]
*C. neoformans* (critical)	Antifungal (MIC = 0.5–8 µg/mL)	-	Additive effects with diamide, H_2_O_2_, fluconazole or AmB ^1^	Assumed targeting of the cryptococcal thioredoxin system	[76]
*C. neoformans* (critical)	Antifungal (MIC = 1–2 µg/mL)	-	-	-	[77]
*C. neoformans* (critical)	Antifungal (MIC = 0.5–4 µg/mL)	Active (8 µg/mL) against *C. neoformans* NR-41292 in *C. elegans*	-	Mia40 inhibition, interference with mitochondrial protein import ^3^	[78]
*C. neoformans* (critical)	Antifungal (MIC_90_ = 2–4 µg/mL)	-	-	-	[83]
*A. fumigatus* (critical)	Antifungal (MIC = 2–4 µg/mL)	-	-	-	[77]
*A. fumigatus* (critical)	Antifungal, fungicidal, antibiofilm	-	Synergy with AmB ^1^ and ITZ ^2^ in ITZ-resistant *A. fumigatus*	SomA and MedA suppression, *Af*TrxR inhibition and downregulation	[84]
*M. mycetomatis* (high)	Antifungal (IC_50_ = 11.2–17.1 µM, IC_90_ = 15.0–45.8 µM)	-	-	-	[87]
*H. capsulatum* (high)	Antifungal (MIC_100_ = 1.25–5 µM), fungicidal (MFC/minimal fungicidal concentration = 2.5–5 µM)	Active (5.7 mg/kg) in infected *T. molitor*	Synergy with AmB ^1^	Reduced fungal cell size, modulation of virulence genes (Hsp70 down, TrxR up)	[93]

^1^ AmB, amphotericin B. ^2^ ITZ, itraconazole. ^3^ mechanistic study using *S. cerevisiae*.

### 4.2. Antifungal Activities of Auranofin–WHO Medium-Priority Group Pathogens and Others

*Candida krusei*, *Cryptococcus gatti*, *Scedosporium* species, *L. prolificans*, and *Coccidioides posadasii* were classified by the WHO as medium-priority fungal pathogens [68]. The pathogens *Candida dubliniensis*, *S. cerevisiae*, *Blastomyces dermatitidis*, and the causative agents of chromoblastomycosis (recorded as a fungal NTD by the WHO) were not listed by now in the WHO priority list but showed considerable susceptibilities to auranofin, which were also described in the following.

Auranofin exhibited high activity against a fluconazole-resistant *C. krusei* isolate and two *C. dubliniensis* strains (Table 2) [75,77]. Thus, together with the data from critical- and high-priority *Candida* pathogens, auranofin has a broad spectrum of anti-*Candida* activity. Mechanistically, the mitochondrial NADH kinase Pos5 was identified as another target of auranofin in yeasts (*S. cerevisiae*), and Pos5 inhibition led to yeast cytotoxicity by cell respiration suppression (Table 2) [80].

Together with *C. neoformans*, *C. gatti* is one of two causative agents of cryptococcosis. In contrast to *C. neoformans*, *C. gatti* was considered to be endemic in Australia, Papua New Guinea, and tropical regions of Africa and South America, but an outbreak in British Columbia and the northwestern USA (Oregon and Washington) in the 2000s as well as the presence of pathogenic *C. gatti* lineages on all continents are alarming and might warrant a higher ranking than only medium-priority for this pathogen [68]. Its global spread beyond endemic (sub-)tropical regions justifies the development of efficient treatment options for *C. gatti* infections. Notably, *C. gatti* was sensitive to auranofin treatment in two different studies, which underscores the promising anti-cryptococcal activities of auranofin (Table 2) [78,83].

*B. dermatitidis* primarily infects the lungs, leading to pneumonia, and is endemic in North America where it overlaps with the endemic regions of histoplasmosis [3]. Auranofin displayed 80% inhibition in three *B. dermatitidis* isolates at concentrations of 1–2 µg/mL and might become an alternative to amphotericin B and azole drugs currently applied for blastomycosis therapy (Table 2) [3,77].

*Scedosporium* species and *L. prolificans* are opportunistic mold pathogens that cause systemic infections in immunocompromised people characterized by a high resistance to the currently available antifungal drugs [3]. Auranofin showed activity against several clinical isolates of *S. apiospermum* and *L. prolificans*. In particular, auranofin was active against the *S. apiospermum* isolate SA-6 (MIC = 4 µg/mL) and six *L. prolificans* isolates, which were resistant to voriconazole (Table 2) [77]. In line with the antifungal activity of auranofin against the drug-resistant molds *S. apiospermum* and *L. prolificans*, a screening of the Pathogen Box library identified auranofin as an active compound against *Scedosporium aurantiacum* (>80% inhibition at 5 µM). Auranofin inhibited the growth of five *Scedosporium* species (*S. aurantiacum*, *Scedosporium boydii*, *S. apiospermum*, *Scedosporium dehoogii*, and *L. prolificans*) with an MIC_80_ value of 5 µM. At this concentration, auranofin suppressed biofilm formation in all five species, and reduced preformed biofilms of *S. aurantiacum* (50% fungal biomass decrease), *S. dehoogii*, and *L. prolificans* (both 70% fungal biomass decrease). Auranofin induced changes in the fungal surface and enhanced the antifungal activities of fluconazole and caspofungin against *S. aurantiacum*. Notably, it exhibited synergy effects in combination with the echinocandin drug caspofungin (Table 2) [94]. In a study using a small panel of five drugs targeting fungal oxidative stress, auranofin and the natural antifungal bis-phenol honokiol (from *Magnolia officinalis*) turned out to be more active than ebselen, PX-12, and conoidin A against 27 *Scedosporium* (*S. apiospermum*, *S. aurantiacum*, *S. boydii*, *S. dehoogii*, *S. minutisporum*) and *L. prolificans* isolates. Auranofin was active against all *Scedosporium* species and *L. prolificans* isolates. In terms of MIC_80_ values (MIC for 80% inhibition), auranofin was also more active than voriconazole and posaconazole against the majority of tested fungal isolates. Additive effects were observed for the combination of auranofin with honokiol in 18 of the 27 isolates. For comparison, only nine isolates exhibited additive effects for the combination of auranofin with voriconazole. Auranofin plus honokiol was most promising in *S. aurantiacum*, *S. boydii*, and *L. prolificans*. The combination of auranofin with sub-lethal doses of the ROS (reactive oxygen species) producer menadione (a 1,4-naphthoquinone derivative) reduced fungal maximal growth, indicating a sensitization of auranofin-treated fungi to oxidative stress. Auranofin displayed a moderate inhibition of *S. apiospermum* TrxR by 26.5% at high doses (32 µg/mL), while honokiol upregulated various oxidoreductase enzymes including peroxiredoxins, thioredoxin reductases, and glutaredoxins in *S. apiospermum* (Table 2) [95]. Together with the previously reported mitochondria-damaging and ROS-producing effects of honokiol in *C. albicans*, the upregulation of oxidoreductases might sensitize *Scedosporium* fungi to the combination of honokiol with auranofin [96].

Chromoblastomycosis is caused by several black fungi (*Cladophialophora*, *Phialophora*, *Exophiala*, *Fonsecaea*, and *Rhinocladiella* species) and is an endemic NTD in (sub-)tropical regions with high case numbers in Mexico (*Fonsecaea pedrosoi*), Brazil (*F. pedrosoi*, *Fonsecaea monophora*), Venezuela (*Cladophialophora carrionii*, *F. pedrosoi*), Madagascar (*C. carrionii*, *F. pedrosoi*), China (*F. pedrosoi*, *F. monophora*, *Fonsecaea nubica*), and Japan (*F. pedrosoi*, *Exophiala* species) [97]. Screening of the Pathogen Box in *F. pedrosoi* revealed a growth inhibition of 82% for auranofin (1 µM) and a MIC_100_ value of 1.25 µM. Tests with a further seven strains confirmed the promising activity of auranofin against chromoblastomycosis fungi (*C. carrionii*, *Phialophora verrucosa*, *Exophiala dermatitidis*, *Exophiala jeanselmei*, *F. monophora*, *F. nubica*, *Rhinocladiella similis*). Moreover, auranofin was fungicidal in two strains (*P. verrucosa* and *E. dermatitidis*) and exhibited synergy effects in combination with itraconazole in *C. carrionii* fungi (Table 2) [98].

Pathogenic *Coccidioides* fungi (*Coccidioides immitis* and *Coccidioides posadasii*) occur in semi-arid regions of North and South America and cause the valley fever (coccidioidomycosis), appearing in the Sonoran and Mojave deserts in the southwest of the USA and the northwest of Mexico [3]. Lopez-Ribot and colleagues recently screened four compound libraries (Broad Institute Repurposing Library, Prestwick Chemicals Library, Selleck L8200 Antiparasitic Library, and MedChemExpress HY-L028 CNS Penetrants Library) for activity against the spherule initials of the *C. posadasii* C735 clinical isolate and identified auranofin as an active compound, albeit it was not among the top compounds (Table 2). For instance, the anthelminthic niclosamide including its ethanolamine salt and the tyrosine kinase inhibitor tyrphostin A9 were active with IC_50_ values below 1 µM [99].

**Table 2:** Antifungal activity of auranofin against medium-priority group pathogens and other species not recorded in the WHO fungal pathogen priority list.

Pathogen(s) (Priority)	Activity		Mechanism(s)	Ref.
	In Vitro	Combinations		
*C. krusei* (medium)	Antifungal (MIC = 0.5 µg/mL)	-	-	[77]
*C. dubliniensis* (not listed)	Antifungal (MIC = 0.62–0.68 µg/mL and MIC_0.3_ = 0.04 µg/mL)	*-*	*-*	[75]
*S. cerevisiae* (not listed)	Fungicidal	-	Pos5 NADH kinase inhibition, suppression of cell respiration	[80]
*C. gatti* (medium)	Antifungal (MIC = 0.5–8 µg/mL)	-	Mia40 inhibition, interference with mitochondrial protein import ^2^	[78]
*C. gatti* (medium)	Antifungal (MIC_90_ = 2 µg/mL)	-	-	[83]
*B. dermatitidis* (not listed)	Antifungal (80% inhibition at 1–2 µg/mL)	-	-	[77]
*S. apiospermum*, *L. prolificans* (medium)	Antifungal (MIC = 1–4 µg/mL for *S. apiospermum* and 2–8 µg/mL for *L. prolificans*)	-	-	[77]
*S. aurantiacum*, *S. boydii*, *S. apiospermum*, *S. dehoogii*, *L. prolificans* (medium)	Antifungal (MIC_80_ = 5 µM), antibiofilm (preformed biofilms of *S. aurantiacum*, *S. dehoogii*, *L. prolificans*)	Synergy with caspofungin (*S. aurantiacum*)	Changes in the fungal surface	[94]
*S. apiospermum*, *S. aurantiacum*, *S. boydii*, *S. dehoogii*, *S. minutisporum*, *L. prolificans* (medium)	Antifungal (MIC = 1–8 µg/mL for *Scedosporium* spp. and 2–8 µg/mL for *L. prolificans*), better MIC_80_ values than voriconazole and posaconazole	Additive effects with honokiol and voriconazole (mainly in *S. aurantiacum* and *L. prolificans*)	Sensitization of fungal cells to ROS, moderate *S. apiospermum* TrxR inhibition	[95]
*C. carrionii*, *E. dermatitidis*, *E. jeanselmei*, *F. pedrosoi*, *F. monophora*, *F. nubica*, *P. verrucosa*, *R. similis* (not listed)	Antifungal (MIC_100_ = 1.25–2.5 µM), fungicidal (MFC = 2.5 µM in *P. verrucosa* and *E. dermatitidis*)	Synergy with ITZ ^1^ in *C. carrionii*	-	[98]
*C. posadasii* (medium)	Antifungal (MIC = 9.54 µM, 6.47 µg/mL)	-	-	[99]

^1^ ITZ, itraconazole. ^2^ Mechanistic study using *S. cerevisiae*.

### 4.3. Critical Evaluation of the Antifungal Activities of Auranofin

The majority of the described studies on the antifungal activities of auranofin are in vitro evaluations. The reported MIC and IC_50_ values were, in many cases, better or at least close to the steady-state mean gold concentration (ca. 3.5 µM after 12 weeks) in the blood of patients undergoing auranofin RA therapy [100]. Certain differences in activity between fungal species and even between the strains of a single species (e.g., among *C. albicans* strains and isolates) were observed. Distinctly higher MIC and IC_50_ values of auranofin were observed in *C. auris*, *C. posadasii*, and *M. mycetomatis*, as well as in certain *C. albicans* strains [81,82,87,99]. But auranofin also exhibited antifungal in vivo activities at higher concentrations in *C. elegans* worm and *T. molitor* insect larvae infection models, as well as antibiofilm activity on catheters used in a murine model [74,78,93]. These tests included *C. auris*-infected *T. molitor*, where auranofin also showed synergy effects with pentamidine [82]. Thus, combination with other drugs can reduce the auranofin concentration required for therapeutic activity. In addition to pentamidine, which increased the uptake of auranofin in *Candida* species, synergy effects were also observed for auranofin combinations with the antimycotic drugs itraconazole (in *A. fumigatus* and *C. carrionii*), amphotericin B (in *A. fumigatus* and *H. capsulatum*), and caspofungin (in *S. aurantiacum*) [84,93,94,98]. All these drugs are targeting vital components of the fungal cell wall and membrane, suggesting a beneficial effect of these mechanisms in combination with auranofin. Moreover, auranofin exhibited distinct antibiofilm activities against *Candida* species, which were superior to the activities of azoles, polyenes, and other antifungal drugs [72]. A detailed discussion of notable verified and hypothetical mechanisms of action of auranofin in fungal diseases is given below (see Section 5.1). Nevertheless, the described approved antifungal drugs are well-established in the clinical therapy of mycoses, which underlines the relevance of the synergy effects of auranofin in combination with these drugs.

It should be noted that the direct comparison of MIC values between different studies is limited by the usage of different criteria and methods. In addition to 50% inhibition, some studies used 80%, 90% or complete (100%) inhibition as criteria for the determination of MIC values. Broth-based microdilutions and optical density analysis were applied for the evaluation of fungal growth inhibition, XTT and resazurin assays were used to investigate the fungal metabolic activity, and XTT, crystal violet, and safranin assays for antibiofilm experiments. Most studies were performed according to the EUCAST (European Committee of Antimicrobial Susceptibility Testing) or CLSI (Clinical and Laboratory Standards Institute) guidelines. Notably, bias was reported as a barrier for successful drug repurposing [101]. But research articles describing the screening of drug libraries usually have a low risk of bias for a specific drug.

An obvious limitation of the antifungal studies about auranofin is the lack of clinical trials with patients suffering from mycoses. The described in vivo antifungal activities of auranofin in worms and insects can be useful for its translation into clinical application as antifungal therapy, together with the data from its decade-long usage as RA therapy and from more recent clinical trials for other infectious diseases such as viral, parasitic, and mycobacterial infections [62,63,64,65,66]. The adverse effects of oral auranofin are well-described and appear to be manageable, but monitoring of patients receiving auranofin therapy is recommended. Diarrhea is reported as the most abundant side effect of oral auranofin therapy, which might require proper treatment adjustments and supporting therapies such as fluid and salt intake by oral and/or intravenous ways. Severe adverse effects such as intra-abdominal sepsis and thrombocytopenia were described as rare complications during auranofin therapy (see Section 3.2) [24,66]. The toxicity of auranofin might be reduced by proper drug formulations, and various nanoparticle carriers have been reported. In terms of gastrointestinal disorders, silk fibroin nanoparticles for auranofin and poly(lactic-co-glycolic) acid-polyethylene glycol (PLGA-PEG) nanoparticles for the precursor complex chlorido(triethylphosphine)gold(I) were disclosed for an efficient drug delivery in colorectal cancers, which conserved the anticancer properties of the gold drugs [102,103]. Moreover, PLGA nanoparticles loaded with auranofin exhibited promising neuroprotective effects in Alzheimer’s disease rat models [104,105]. This formulation might become of importance for the treatment of brain-infecting fungi such as *C. neoformans* with auranofin. A topical formulation based on auranofin-loaded nanoparticles and a thermo-responsive hydrogel was also reported for the treatment of intravaginal trichomonad infections, and the formulated auranofin was superior to oral auranofin in mice in terms of activity and toxicity [106]. Thus, formulated auranofin might also be of interest for the topical therapy of vaginal mycoses such as vulvovaginal candidiasis where new alternative treatments are required [107].

Suitable formulations of auranofin can be beneficial for its development as an antifungal drug. But the repurposing of a drug is challenging and can pose several unexpected problems that should be considered in addition to the advantages of drug repurposing. In addition to efficacy and safety issues, economic and legal reasons can hamper the development [101]. Clinical trials with anti-infective drugs often fail not only because of limited efficacy and safety but also because of problems with the study design, the complexity of the studied microorganism(s) and the associated disease manifestations (including host responses), and the recruiting of enough suitable patients [108]. Furthermore, the properties of the repurposed drug should be compared with those of antifungals that were recently approved or are currently in late stages of clinical development.

The intravenously administered second-generation echinocandin and 1,3-β-glucan synthase inhibitor rezafungin was approved in 2023 for the therapy of candidemia and invasive candidiasis and displayed promising activities in clinical trials with *Candida* species [109]. It is a caspofungin analog with improved pharmacokinetics (prolonged half-life) and reduced hepatotoxicity showing broad activity against *Candida* and *Aspergillus* pathogens while being inactive, in line with other enchinocandins, against *C. neoformans*, *Trichosporon* species, and non-*Aspergillus* molds [110,111]. Thus, rezafungin lacks the anti-cryptococcal activity of auranofin. The triterpenoid drug ibrexafungerp is a first-in-class oral inhibitor of 1,3-β-glucan synthase with a different enzyme binding site than echinocandins. It is approved for the therapy of vulvovaginal candidiasis and shows activity against various echinocandin-resistant *Candida* strains [110,111]. But like echinocandins, ibrexafungerp also lacks activity against *C. neoformans*. Since caspofungin exhibited synergy effects with auranofin in *S. aurantiacum*, rezafungin and ibrexafungerp might be promising and safer combination partners for auranofin in *Scedosporium* species [94]. Based on the activity spectrum of rezafungin and ibrexafungerp, combinations of auranofin with these new 1,3-β-glucan synthase inhibitors might also be reasonable for the treatment of critical-priority pathogens such as *C. albicans*, *C. auris*, and *A. fumigatus*. The experimental phosphate prodrug fosmanogepix (i.e., the prodrug of the pyridine-isoxazole derivative manogepix) is a first-in-class GPI (glycosylphosphatidylinositol) anchor inhibitor of the enzyme Gwt1 (glycosylphosphatidylinositol-anchored wall protein transfer 1) and suppresses inositol acylation required for the function of GPI-anchored mannoproteins in the cell wall. It has a low cross-resistance with azoles and echinocandins, and fosmanogepix is also active against *Cryptococcus* species [110,111]. But it was reported that *C. krusei* is resistant to fosmanogepix, and thus auranofin has the potential to outperform fosmanogepix in *C. krusei* infections. The experimental DHODH inhibitor olorofim is a first-in-class drug of the orotomide class of antifungals, which suppresses pyrimidine biosynthesis. It is a more specific antifungal showing promising activity against *Aspergillus*, *Coccidioides*, *Histoplasma*, *Scedosporium*, and *Madurella* species, but it remained inactive against *Candida* and *Cryptococcus* species as well as against *F. pedrosoi* [110,111]. Hence, auranofin shows a broader antifungal spectrum than olorofin and appears to be superior in chromoblastomycoses as well as in *Candida* and *Cryptococcus* pathogens.

## 5. Antifungal Mechanisms of Auranofin and Development of New Gold Complexes

### 5.1. Auranofin Antifungal Targets and Mechanisms of Action

The antifungal mechanisms of auranofin were briefly described in the above sections. The scope and a broader discussion of the mentioned modes of action, as well as a selection of auranofin mechanisms from other diseases, which might become relevant for antifungal drug discovery, are provided below.

#### 5.1.1. Thioredoxin Reductase and ROS

**Figure 2:**
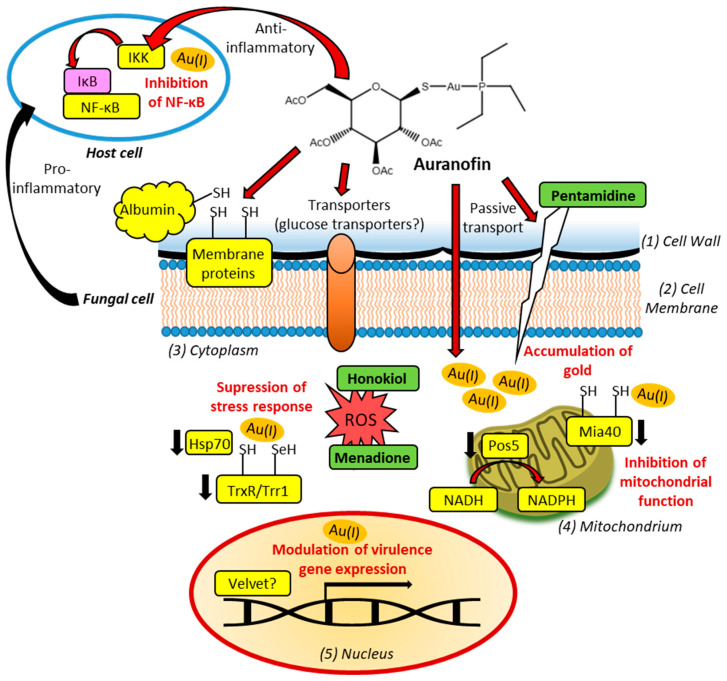
Confirmed and proposed mechanisms of auranofin and its metabolites such as (triethylphosphine)gold(I) and aurocyanide, symbolized as golden Au(I) icons, to fight fungal infections. Uptake of auranofin can occur actively by transporters or membrane proteins in cooperation with albumin or passively. Pentamidine disrupts fungal cell walls (1) and membranes (2), leading to increased gold uptake in auranofin-treated fungal cells [80,81]. Auranofin suppresses stress response by inhibition of TrxR proteins [in the cytoplasm (3) or mitochondrion (4)] and downregulation of Hsp70 [76,84,93,95]. Activities of ROS-producing agents such as honokiol and menadione are augmented by auranofin [95]. Moreover, auranofin inhibits mitochondrial targets (4) such as Mia40 involved in protein import and the NADH kinase Pos5 [78,80]. As an IKK inhibitor, auranofin can suppress NF-κB signaling in host cells and reduce inflammation [59,162,163,164]. Fungal velvet proteins with a DNA-binding domain similar to NF-κB might be a new auranofin target in the nucleus (5) of fungi [169,170,171].

The cytotoxic properties of auranofin were often associated with inhibition of TrxR. This NADPH-dependent selenoprotein is crucial for the reduction and reactivation of thioredoxin as part of the thioredoxin system, and thus for the maintenance of cell redox homeostasis [112]. Imbalances in this system lead to the accumulation of cytotoxic ROS. Auranofin showed TrxR-inhibitory activities in *A. fumigatus* and *S. apiospermum*, while hints at auranofin-mediated TrxR inhibition were also reported in *C. neoformans* [76,84,95]. However, the TrxR inhibition in *S. apiospermum* was only moderate. Moreover, TrxR gene regulation by auranofin was also documented in treated fungi (e.g., suppression in *A. fumigatus*, upregulation in *H. capsulatum*) [84,93]. It was shown that auranofin sensitized fungal cells to other ROS-producing compounds such as menadione and honokiol, probably by TrxR inhibition (Figure 2) [95].

In *S. cerevisiae*, the fungal TrxR1 homolog Trr1 (thioredoxin reductase 1) is located in the cytoplasm, while Trr2 is found in mitochondria. Trr1-deleted *S. cerevisiae* turned out to be hypersensitive to H_2_O_2_ and elevated temperatures [113,114,115]. In silico evaluations suggest Trr1 to be essential in eight different pathogenic fungi (*C. albicans*, *A. fumigatus*, *B. dermatitidis*, *Paracoccidioides brasiliensis*, *Paracoccidioides lutzii*, *C. immitis*, *C. neoformans*, and *H. capsulatum*) and thus Trr1 can be a promising target for broad-spectrum antifungal agents in yeasts, molds, and dimorphic pathogenic fungi [116]. The vital role of Trr1 was confirmed in *C. neoformans* (preferentially located in the mitochondria here) and *Paracoccidioides* species, as well as in *A. fumigatus* (denoted as *Af*TrxR) [117,118,119,120]. Notably, upregulated thioredoxin is crucial for the development of *C. albicans* biofilms [121]. Trr1 was used as a target to screen possible antifungal compounds against *C. neoformans*, *Paracoccidioides* species, and *A. fumigatus* (*Af*TrxR) [117,121,122]. In addition to auranofin, other well-established TrxR inhibitors such as ebselen and PX-12 exhibited antifungal activities [115,123]. The antimicrobial properties of crystal violet, which was applied as an antibiotic drug in the first half of the 20th century, were attributed to TrxR2 inhibition [124]. Since Trr1 was identified as an immunogenic antigen on the cell surfaces of *C. albicans*, *C. neoformans*, and *Paracoccidioides* species, it is possible to develop therapeutic antibodies against the surface Trr1 proteins of these fungi [125].

Because mammalian TrxR enzymes were also inhibited by auranofin, it remains unclear how far fungal TrxR proteins can be a selective target for auranofin. Inhibition of mammalian TrxR by auranofin was found to be variable depending on the applied model and incubation times, and inhibitory concentrations were between the low nanomolar and micromolar concentration range. Auranofin showed a high inhibition of TrxR from human placenta and rats [126,127]. In cancer cells, high activities of auranofin were observed in lymphoma, myeloma, and ovarian cancer cells, while lower activities were found in melanoma, breast carcinoma, and hepatocellular carcinoma cells [128,129,130,131,132]. Thus, it is difficult to compare the activities of auranofin against fungal TrxR with those from mammalian cells. Trialkyl- and triphenylphosphine ligands appear to play an important role, and it was shown that such phosphine ligands in auranofin-type complexes were superior to gold(I) complex analogs with arsine and stibine ligands in terms of TrxR inhibition [133].

#### 5.1.2. Mitochondrial Targets in Yeasts

There is evidence that TrxR proteins are not the primary antimicrobial targets of auranofin in bacteria, and it was shown that auranofin can also attack other important proteins [134]. As in virtually all eukaryotes, mitochondria also play a vital role in cells of human pathogenic fungi, and the disturbance of mitochondrial function offers a valuable fungal drug target [135]. It was shown that auranofin can inhibit essential mitochondrial proteins such as the oxidoreductase Mia40 and the NADH kinase Pos5 in yeasts (Figure 2) [78,80]. Auranofin disturbs the interaction of Mia40 with substrates in the mitochondrial intermembrane space (IMS) and consequently blocks mitochondrial protein import via the Mia40-Erv1 pathway (mitochondrial disulfide relay) [78]. Fungal Mia40 has an N-terminal hydrophobic anchor for the mitochondrial inner membrane and a large C-terminal domain with invariant Cys residues exposed to the IMS. Unfolded substrates (pre-proteins) translocated to the IMS from the cytoplasm (via the Tom complex) are recognized (via the substrate MISS motif) and trapped by Mia40 in the IMS by formation of mixed disulfide bonds with the reduced substrates. In the following, Mia40 folds the bound substrate by the introduction of disulfide bonds (two disulfides in most substrates) before the folded substrate is released from Mia40 mediated by the associated sulfhydryl oxidase Erv1 and the zinc-binding protein Hot13. The list of known Mia40 substrates underlines the importance of Mia40 for functional mitochondria and its promising role as a drug target. It includes small Tim (translocase of the inner membrane) proteins (Tim8-10, Tim12, Tim13), the superoxide dismutase Sod1 and its copper chaperone Ccs1, the protein stabilizer Mdm35, proteins involved in respiration (Cox12, Qcr6, Rip1), and factors of copper transport and complex IV assembly (Cox17, Cox19, Cmc1, Cmc2, Som1) [136]. The catalytic dithiol of Mia40 forms long-lived mixed disulfides with substrates and is surrounded by a hydrophobic groove [137,138]. Thus, it is possible that the hydrophobic character of the triethylphosphine ligand of auranofin plays a crucial role in the interaction of this gold drug with Mia40. Notably, the trialkyl/triaryl-substituted phosphine ligand in gold(I) complexes is required for biological activity [53]. Moreover, the phosphine ligand could contribute to mitochondria targeting of auranofin since cationic gold complexes with lipophilic character can accumulate in mitochondria, and the triphenylphosphonium moiety is a well-established targeting device for mitochondria [139].

Pos5 is a mitochondrial NADH kinase, which was identified as another target of auranofin in *S. cerevisiae*. Inhibition of Pos5 by auranofin suppressed cell respiration, ultimately leading to yeast cell death [80]. Pos5 is located in the mitochondrial matrix of yeasts and phosphorylates NADH to NADPH, which is required for antioxidant activity, mitochondrial iron homeostasis, and arginine biosynthesis [140]. Pos5 maintains stability of mitochondrial DNA by inactivation of ROS, while Pos5 deletion enhanced oxidative damage tremendously and led to endogenous copper hypersensitivity [141]. Moreover, Pos5 is necessary for functional respiratory chain complexes and involved in mitochondrial Fe-S cluster synthesis [142,143]. Pos5 was also shown to be relevant for *Candida viswanathii* growth and for virulent polysaccharide capsule production by *C. neoformans* [144,145]. Thus, together with its Mia40 inhibitory activity, auranofin attacks crucial mitochondrial factors and pathways in yeasts. Notably, another mitochondria-targeting drug, the anthelminthic niclosamide, was repurposed as an antifungal agent and exhibited promising activities against several pathogenic fungi. The mitochondria-damaging mechanisms of niclosamide include disruption of oxidative phosphorylation and suppressed ATP synthesis (niclosamide is a protonophor), inhibition of Mge1 (nucleotide-exchange factor associated with the mitochondrial Hsp70 protein Ssc1 and the Tim23-Tim17 mitochondrial protein import complex), inhibition of NDU1 (NADH ubiquinone oxidoreductase complex 1 of the inner membrane), and suppression of mitochondrial oxygen consumption [146]. These mechanisms add well to those described for auranofin, and combinations of niclosamide with auranofin might be promising for the therapy of pathogenic yeasts. How far the described mitochondrial yeast targets of auranofin are also relevant for the treatment of molds and dimorphic pathogenic fungi remains to be elucidated.

#### 5.1.3. Chaperones

The Mia40-mediated effects on mitochondrial chaperones (Ccs1) and protein stabilizers (Mdm35) provide hints at an influence of auranofin on protein homeostasis. The copper chaperone Ccs1 is associated with Sod1, while Mdm35 stabilizes Ups1 and Ups2 (ubiquitin-proteasome system 1 and 2) in the IMS [136]. Sod1, as well as the Hsp70 and Hsp90 chaperone proteins, are required for yeast stress response [147]. Moreover, fungal heat-shock proteins are involved in morphological transitions (e.g., mycelia to yeast) and antifungal drug resistance in fungal pathogens [148]. Hsp70, Hsp90, and other heat-shock proteins (e.g., Hsp12 and Hsp40) also play a vital role in *Aspergillus* molds [149]. Notably, the treatment with auranofin suppressed virulent Hsp70 expression in *H. capsulatum* (Figure 2) [93]. Synthetic (CMLD013075) and natural (EGCG) inhibitors of fungal Hsp90 showed promising activities against *Candida* species [10,150]. In addition, Hsp70 played a central role in the Hsp90-mediated tolerance of *A. fumigatus* to caspofungin [151]. Thus, the targeting of fungal heat-shock proteins appears to be a promising strategy in yeasts, molds, and dimorphic fungi. Upregulated Hsp70 expression was frequently observed as a consequence of Hsp90 inhibition, which renders Hsp70 a valuable drug target [152]. Compounds such as auranofin, which can downregulate Hsp70, might be beneficial for Hsp90-targeting therapies.

*C. albicans* can also build an extracellular form of Hsp90 (eHsp90), which is responsible for increased *C. albicans* virulence. The virulence of eHsp90 is mediated by upregulated NF-κB signaling, leading to pyroptosis of macrophages [153]. The possible role of NF-κB as a host target of auranofin in mycotic infections is discussed below.

#### 5.1.4. Host-Directed Anti-Inflammatory Effects–Hypothetic Targets for Antifungal Auranofin

The host transcription factor NF-κB is responsible for a strongly increased inflammation in many infectious diseases, and a prominent example is the NF-κB-mediated hyperinflammation (also known as. cytokine storm) in COVID-19 patients [154]. In terms of fungal infections, *C. albicans* induced inflammation in keratinocytes by upregulation of NF-κB, while *A. fumigatus* led to hyperinflammation in a cystic fibrosis mouse model by increased NF-κB signaling [155,156]. In addition to paracrine effects on adjacent host cells, certain fungal pathogens (e.g., *C. albicans*, *C. neoformans*, *A. fumigatus*, *C. immitis*, and *H. capsulatum*) can infect and replicate in host cells such as macrophages [157]. Notably, cells infected with *C. neoformans* showed upregulated NF-κB, leading to fungal-induced cell apoptosis [158]. Thus, NF-κB is a promising host target for antifungal agents to reduce fungal virulence. *A. fumigatus*-infected mice fed with vitamin D had higher survival rates than vitamin D-deficient mice, and lower NF-κB expression levels were observed in the macrophages of mice treated with vitamin D [159]. Notably, auranofin was able to augment the activity of 1α,25-dihydroxyvitamin D_3_ in leukemia cells [160]. Auranofin can bind to IKK, which ultimately leads to NF-κB inhibition (see Section 3.2, Figure 2) [59]. Whether auranofin reduces fungal virulence and associated host inflammation processes by inactivation of NF-κB remains to be elucidated. However, it was shown that anti-inflammatory agents such as thymol can suppress keratitis by *A. fumigatus* infection via downregulation of NF-κB signaling [161]. Since auranofin also showed beneficial effects in liver disease models and inhibited hepatic steatosis and fibrosis in NAFLD (non-alcoholic fatty liver disease) by suppression of NF-κB activity, it might also ameliorate the liver-damaging effects of the approved antifungals flucytosine and amphotericin B [162,163]. The combination of auranofin with amphotericin B displayed synergy effects in various pathogenic fungi (see Section 4.1 and Section 4.2), and thus, this therapy option might also reduce the risk of drug-mediated hepatotoxicities. Moreover, auranofin prevented osteoporosis by suppression of the RANKL (receptor activator of NF-κB ligand)/NF-κB signaling pathway and associated osteoclastogenesis [164]. Activated RANKL signaling was correlated with bone damage in various infectious diseases [165]. Bone infection and damage were also observed in several mycoses, including eumycetoma, which can require amputation in severe, advanced cases [166,167]. Notably, the RANKL pathway was upregulated by *C. albicans* in an oral candidiasis mouse model [168]. Thus, auranofin might also be considered for the suppression and prevention of bone-damaging inflammation processes during fungal infections.

Although fungi do not have NF-κB, the fungal velvet proteins (VeA, VelB, and VelC) have a DNA-binding domain structurally similar to the one of animal NF-κB (Figure 2) [169]. The velvet proteins are important factors for the regulation of fungal development and secondary metabolism [170]. Since no fungal IκB homolog has been identified so far, the regulation of velvet proteins obviously differs from NF-κB, but various phosphorylation sites of VeA indicate a vital role of VeA-interacting protein kinases (e.g., FphA, MpkB) for the modulation of VeA activity [171]. Whether one of these velvet-regulating fungal protein kinases can be targeted by auranofin analogously to IKK remains to be shown. Moreover, cinnamaldehyde efficiently suppressed the biosynthesis of the notorious aflatoxin in *A. flavus* via modulation of the expression of velvet complex proteins [172,173]. Analogously, 2-hydroxy-4-methoxybenzaldehyde inhibited the formation of the toxic secondary metabolite deoxynivalenol in *Fusarium graminearum* [174]. Thus, velvet proteins appear to be a promising target for antifungal drugs.

#### 5.1.5. Drug Uptake in Fungal Cells–Opportunity for Combination Therapies

The mechanisms of the cellular uptake of metal-based drugs have been thoroughly studied and can include active (via transporters and endocytosis processes) and passive ways (neutral, hydrophobic complexes can cross cell membranes). The hydrophobic character of auranofin suggests a considerable passive transport across membrane bilayers, while the thiosugar ligand of auranofin might enable active transport via glucose transporters [175]. Apart from a possible transmembrane transport of carbohydrate-based drugs, fungal glucose transporters have emerged as new antifungal drug targets in their own right [176]. Considering the liability of the thiosugar ligand, a “thiol-shuttle” model via consecutive thiol ligand exchange involving serum albumin and membrane sulfhydryl proteins was also discussed for auranofin (Figure 2) [62,177]. In addition, the formation of the [Au(CN)_2_]^-^ metabolite enhanced gold uptake in red blood cells [62,178]. The degree of intracellular auranofin accumulation can have an influence on its antifungal activities. The cell membrane permeability of *C. albicans* was increased by pentamidine, which led to a 100% higher cellular uptake of auranofin, accompanied by distinctly higher antifungal activity [81]. In addition to *C. albicans*, pentamidine led to leaky membranes and exhibited synergy effects together with auranofin in *C. auris* [82]. A pentamidine-mediated cell wall- and membrane-disrupting effect in association with a higher auranofin uptake was also observed in Gram-negative bacteria, and both drugs were listed as suitable treatments to overcome antibiotic resistance [179,180]. In addition to increased cell wall and membrane permeability, pentamidine could also inhibit drug efflux pumps in Gram-negative bacteria, which contributed to its synergy effect with antibiotics [181]. Since the antiparasitic drug pentamidine is also clinically applied for the treatment of *Pneumocystis jirovecii*-mediated pneumonia in immunocompromised persons (e.g., HIV patients) and was active against various other pathogenic fungi (e.g., *Sporothrix schenckii*, *Fusarium* species, *C. posadasii*), a combination with auranofin might also be promising in these species to enhance the antimycotic efficacies of both drugs, best in a synergistic way [99,182,183,184]. The described synergy effects of auranofin with the approved cell wall- and membrane-damaging/interfering antifungals amphotericin B (in *C. albicans*, *A. fumigatus*, and *H. capsulatum*), caspofungin (in *S. aurantiacum*), and itraconazole (*A. fumigatus*, *C. carrionii*) support the promising treatment strategy to combine auranofin with drugs targeting fungal cell walls and membranes (see also Section 4.3) [73,84,93,94,98]. Based on the poly-cationic character of pentamidine under physiological pH, which enables the interaction with negatively charged microbial cell wall components and the penetration of cell walls and membranes, various poly-nitrogenous derivatives with improved Gram-negative bacterial cell wall-targeting properties were described as promising adjuvants for antibiotic drugs [185,186]. These new pentamidine analogs might also be suitable combination partners for auranofin in bacteria and fungi.

### 5.2. Emerging Antifungal Gold Complexes

**Figure 3:**
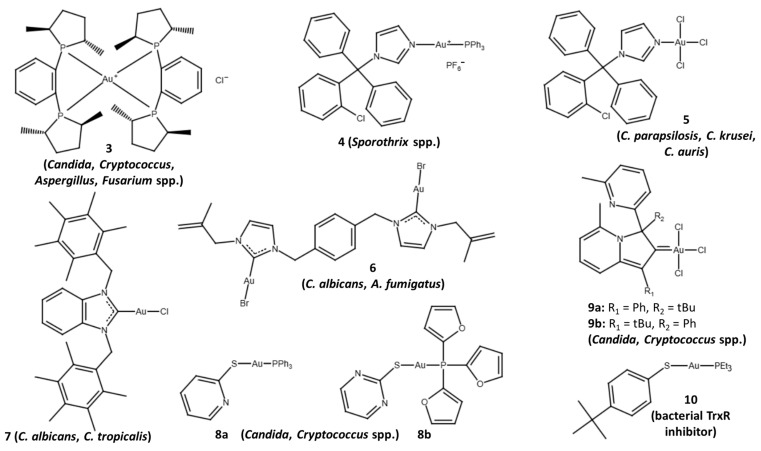
Structures of gold complexes with antifungal potential (sensitive fungi in brackets for complexes **3–9** and bacterial target in brackets for complex **10**): cationic bis-phosphine complex **3**, clotrimazole complexes **4** and **5**, NHC complexes **6** and **7**, auranofin-type complexes **8a** and **8b** with pyridine-2-thiol and pyrimidine-2-thiol ligands, carbene complex isomers **9a** and **9b**, and antibacterial auranofin-type TrxR inhibitor **10** with 4-*tert*-butylbenzenethiol ligand.

Based on the pronounced biological activities of auranofin, continuous research efforts deal with the design of new and more active gold complexes. A selection of promising antifungal gold complexes is given below (Figure 3).

The antimycotic properties of complexes **1** and **2** in critical- and high-priority pathogens were already described (see Section 4.1 and Figure 1). But complex **1** also showed antifungal activities against *A. niger* (MIC = 15.63 µg/mL), while **2** was active against *A. niger* and *C. krusei* (MIC = 10–25 µg/mL) [70,71]. In particular, the example of complex **1** shows that the thiosugar ligand of auranofin can be replaced by other thiol derivatives, including ligands with intrinsic biological activities. The quinolizidine alkaloid-derived lupinylsulfide ligand of **1** led to higher blood gold levels by this complex in arthritic rats in comparison with auranofin-treated animals [187]. The miscellaneous bioactivities of quinolizidine alkaloids including lupinine-type derivatives were reviewed recently and might be beneficial in combination with gold-mediated effects [188].

The modification of the phosphine ligand system proved to be a promising strategy for the design of potent gold antifungals. Garneau-Tsodikova and coworkers designed a series of distorted gold(I)-phosphine complexes, and the square-planar complex **3** with two phenyl-bridged bis-phosphine ligands exhibited broad-spectrum activity against *Candida*, *Cryptococcus*, *Aspergillus*, and *Fusarium* species, including multidrug-resistant *C. auris* strains (Figure 3). Notably, complex **3** showed higher antibiofilm activities against *C. auris* strains than auranofin and a relatively low toxicity to mammalian cells [189].

Antifungal azoles are *N*-heterocycles and thus can be applied as *N*-ligands for bioactive transition metal complexes [190]. Various gold complexes with azole drug ligands such as ketoconazole (KTZ) and clotrimazole (CTZ) were described. The cationic (triphenylphosphine)gold(I) complexes [Au(PPh_3_)(KTZ)]PF_6_ and [Au(PPh_3_)(CTZ)]PF_6_ **4** exhibited high (i.e., nanomolar) fungal growth inhibitory and fungicidal activities against *Sporothrix* species (sporotrichosis is listed as a fungal skin NTD by the WHO), as well as considerable selectivity for these fungi (Figure 3). Both complexes modified fungal cell morphology and outperformed the antifungal activities of the metal-free azole drugs (KTZ, CTZ) [191]. Trichloridogold(III) complexes of the AuCl_3_(azole) type were also prepared and investigated for their activity against various *Candida* species. Notably, AuCl_3_(CTZ) **5** was active against *C. auris* (IC_50_ = 1.9 µM), *C. parapsilosis* (IC_50_ = 1.4 µM), and *C. krusei* (IC_50_ = 0.4 µM) (Figure 3) [192].

Certain azoles such as imidazoles and benzimidazoles can be applied for the synthesis of *N*-heterocyclic carbene/NHC-metal complexes. Originally developed as versatile catalysts in synthetic organic chemistry, the medicinal relevance of NHC-metal complexes is growing continuously. Gold-NHC complexes were frequently described as anticancer, antiviral, and antiparasitic agents [40,193,194]. A dinuclear phenyl-bridged bis-imidazolylidene gold(I) complex **6** with bromido ligands was active against *C. albicans* (MIC = 1.95 µM) and *A. fumigatus* (MIC = 0.97 µM), which was comparable with amphotericin B activity and superior to fluconazole activity against these fungi (Figure 3). In addition, the antibiofilm activity of this complex against both fungal strains was superior to amphotericin B and fluconazole, and the complex induced membrane damage in *C. albicans* cells associated with leakage of cytoplasmic material [195]. A Turkish group disclosed a structurally simple chlorido-[1,3-bis(2,3,4,5,6-pentamethylbenzyl)benzimidazol-2-ylidene]gold(I) complex **7** with antifungal activity against *C. albicans* and *C. tropicalis* (MIC = 12.5 µg/mL in both fungi) (Figure 3) [196].

A library of metal compounds was established by the Community for Open Antimicrobial Drug Discovery (CO-ADD) and tested for antifungal activity against *C. albicans* and *C. neoformans*. Two gold(I) complexes **8a** and **8b**, both linear auranofin-type complexes with thiol (pyridine-2-thiol for **8a**, and pyrimidine-2-thiol for **8b**) and phosphine ligands (triphenylphosphine for **8a** and tri(2-furyl)phosphine for **8b**), and a trichloridogold(III)-carbene complex **9** (mixture of isomers **9a** and **9b**, their carbene ligands are derived from a gold-mediated cyclization of a bispyridylallene precursor) exhibited high activity against *C. neoformans* (MIC = 0.024–0.195 µM) (Figure 3). **8a** was also active against *C. albicans*, *C. glabrata*, *C. tropicalis*, two *C. auris* strains, and two *C. deuterogatti* strains with nanomolar MIC values (0.024–0.781 µM). Notably, the carbene complex **9** was highly active against the *C. auris* CBS12373 strain (MIC ≤ 0.006–0.049 µM) and the *C. deuterogatti* ATCC 32609 strain (MIC = 0.024–0.049 µM). While **8b** showed toxicity in *G. mellonella* larvae at 1 mM, both **8a** and **9** were non-toxic at this concentration. However, the gold complexes did not prolong the lives of larvae infected with *C. albicans* [197].

It should be noted that numerous TrxR-inhibitory gold complexes were developed as new anticancer drug candidates, which were reviewed recently [198]. The most recent progress covered the design and development of gold-based dual TrxR/receptor tyrosine kinase (EGFR) inhibitors [199,200]. How far these cytotoxic gold compounds can become suitable for antifungal therapies remains to be shown. However, gold-based TrxR inhibitors, which were selective for bacterial microbes, might be promising drug candidates for the therapy of fungal infections, too. In particular, the new bacterial TrxR-targeting auranofin derivative **10** with a 4-*tert*-butyl-benzenethiol ligand derived from the known NF-κB and TrxR inhibitor BAY-117085 turned out to be an efficient antibiotic in *S. aureus*, *Enterococcus faecium*, and *Helicobacter pylori* strains without hemolytic properties (Figure 3) [201]. Whether complex **10** also outperforms auranofin in fungal pathogens remains to be shown.

Thus, the design of new antifungal gold compounds included both linear and square-planar Au(I) and Au(III) complexes. The chemistry of gold ions with the common oxidation states +I and +III in aqueous systems is well-elaborated. In vivo, the predominant oxidation state is +I, and it is assumed that Au(III) ions are reduced to Au(I) in organisms [24,28]. Notably, the composition of most active gold complexes including auranofin is a coordination of the gold(I) or gold(III) center to one or more exchangeable ligands (e.g., halides or sulfides) and a more stably coordinated spectator ligand (phosphine, nitrogen, or carbene ligands, including chelate ligands). In the case of auranofin it was shown that the metal-free ligands (triethylphosphine and 2,3,4,6-tetraacetyl-1-thio-β-D-glucose) have no considerable bioactivities [53,54]. The (triethylphosphine)gold(I) complex fragment of auranofin was shown to be crucial for its antitoxoplasmal activity since other phosphine-gold(I) complexes also exhibited high activities against *Toxoplasma gondii* parasites [53]. The exchange of the labile ligand followed by reaction with bionucleophiles, similar to bioactive platinum(II) complexes such as cisplatin, is assumed to play a role due to their activity together with the lipophilic character of the spectator ligand. However, as in the case of cisplatin, the spectator ligand of gold complexes can also be exchanged in the presence of thiols. In terms of auranofin, albumin and thiols could replace the triethylphosphine ligand bound to the Au(I) center, and the released phosphine ligand was irreversibly converted to triethylphosphine oxide by disulfide-mediated oxidation, thus preventing the reverse reaction of gold(I) with the released phosphine molecule [202].

In addition to auranofin-type complexes, delocalized lipophilic cations (DLCs) form another group of promising gold complex antifungals, which is exemplified by the potent complexes **3** and **4**. It is noteworthy that DLCs have the property to accumulate in mitochondria, which is assumed to be a preferred biological target of DLCs [175]. The relevance of fungal mitochondria-targeting by auranofin was outlined above (see Section 5.1).

## 6. Conclusions

The orally applicable drug auranofin can become a new salient drug for various problematic fungal infections. Its activity spectrum comprises growth inhibitory and fungicidal properties, as well as inhibition of fungal and mixed bacterial/fungal biofilms. The modes of action of auranofin include inhibition of thioredoxin reductase and of vital mitochondrial targets, which have synergy potential with the mechanisms of currently available antimycotics (e.g., azoles, amphotericin B, caspofungin) and other drugs under antifungal investigation such as the antiparasitic agent pentamidine and the natural phenol honokiol. Thus, auranofin might prevent and overcome antifungal drug resistance. In addition, the well-established anti-inflammatory activities of auranofin have the potential to mitigate fungal virulence in infected organisms, which can be beneficial for the healing process. However, the influence of the immunomodulatory properties of auranofin on fungal infections remains to be elucidated in more detail, also because many mycoses mainly emerge in immunocompromised patients. Nevertheless, the described in vivo activities of auranofin (*C. neoformans*-infected *C. elegans*, *H. capsulatum*-infected *T. molitor*, and *C. auris*-infected *T. molitor*) are promising and might be confirmed in mammalian infection models. Adverse effects of auranofin in humans are usually mild; however, the casual appearance of life-threatening complications such as abdominal sepsis requires close monitoring of patients receiving auranofin therapy. But according to the currently available clinical and pharmacological data of auranofin, the development of a new gold-based fungicide appears to be possible and desirable. Further studies in mammalian infection models and clinical trials are needed to validate auranofin’s antifungal efficacy and safety in humans.

## Data Availability

Antifungal activities, including original data, of auranofin against *M. mycetomatis* can also be found on the Mycetoma Open Source/MycetOS website, https://github.com/OpenSourceMycetoma (accessed on 12 April 2025).

## Supplementary Materials

The supporting information can be downloaded at https://www.mdpi.com/article/10.3390/ijms26167909/s1.

## Abbreviations

The following abbreviations are used in this manuscript:

AmB	Amphotericin B
ART	Antiretroviral therapy
CLSI	Clinical and Laboratory Standards Institute
CO-ADD	Community for Open Antimicrobial Drug Discovery
COX-2	Cyclooxygenase-2
CTZ	Clotrimazole
DHODH	Dihydroorotate dehydrogenase
DLC	Delocalized lipophilic cation
DNDi	Drugs for Neglected Diseases initiative
Erv1	Essential for respiratory growth and viability 1
EUCAST	European Committee of Antimicrobial Susceptibility Testing
GPI	Glycosylphosphatidylinositol
Gwt1	Glycosylphosphatidylinositol-anchored wall protein transfer 1
HDAC	Histone deacetylase
Hsp	Heat-shock protein
IKK	IκB kinase
IMS	Intermembrane space (in mitochondria)
ITZ	Itraconazole
KTZ	Ketoconazole
MIC	Minimal inhibitory concentration (usually for inhibition of ≥ 50%)
MIC80	MIC for ≥ 80% inhibition
MIC90	MIC for 90% inhibition
MIC100	MIC for 100% inhibition
MIC0.3	MIC for ≥ 30% inhibition
MFC	Minimal fungicidal concentration
Mia40	Mitochondrial IMS import and assembly pathway 40 kDa
MMV	Medicines for Malaria Venture
MycetOS	Mycetoma Open Science
NAFLD	Non-alcoholic fatty liver disease
NHC	N-heterocyclic carbene
NF-κB	Nuclear factor-κB
NK cells	Natural killer cells
NOS	Nitric oxide synthase
Nrf2	Nuclear factor erythroid 2-related factor 2
NTD	Neglected tropical disease
PEG	Polyethylene glycol
PLGA	Poly(lactic-co-glycolic) acid
Pos5	Peroxide sensitive 5
PU	Polyurethane
RA	Rheumatoid arthritis
RANKL	Receptor activator of NF-κB ligand
ROS	Reactive oxygen species
Tim	Translocase of the inner membrane
Tom	Translocase of the outer membrane
Trr	Thioredoxin reductase (in fungi)
TrxR	Thioredoxin reductase (general)
UPS	Ubiquitin-proteasome system
WHO	World Health Organization
