# Supplementary material for: Exploring a Therapeutic Gold Mine: The Antifungal Potential of the Gold-Based Antirheumatic Drug Auranofin
Source: Int J Mol Sci. 2025 Aug 16;26(16):7909. doi: 10.3390/ijms26167909 (PMC12386961; doi:10.3390/ijms26167909)
Supplement: Supplementary file 1 [file ijms-26-07909-s001.zip › ijms-3752346-supplementary.pdf]

Supplementary Materials

Exploring a Therapeutic Gold Mine: The Antifungal Potential of the Gold-Based Antirheumatic Drug Auranofin

Jingyi Ma [1], Wendy van de Sande [1] and Bernhard Biersack [2,*]

[1] Department of Medical Microbiology and Infectious Diseases, Erasmus MC, University Medical Center Rotterdam, Dr. Molewaterplein 40, 3015 GD Rotterdam, The Netherlands
[2] Organic Chemistry Laboratory; University of Bayreuth, Universitätsstrasse 30, 95440 Bayreuth, Germany

* Correspondence: bernhard.biersack@yahoo.com

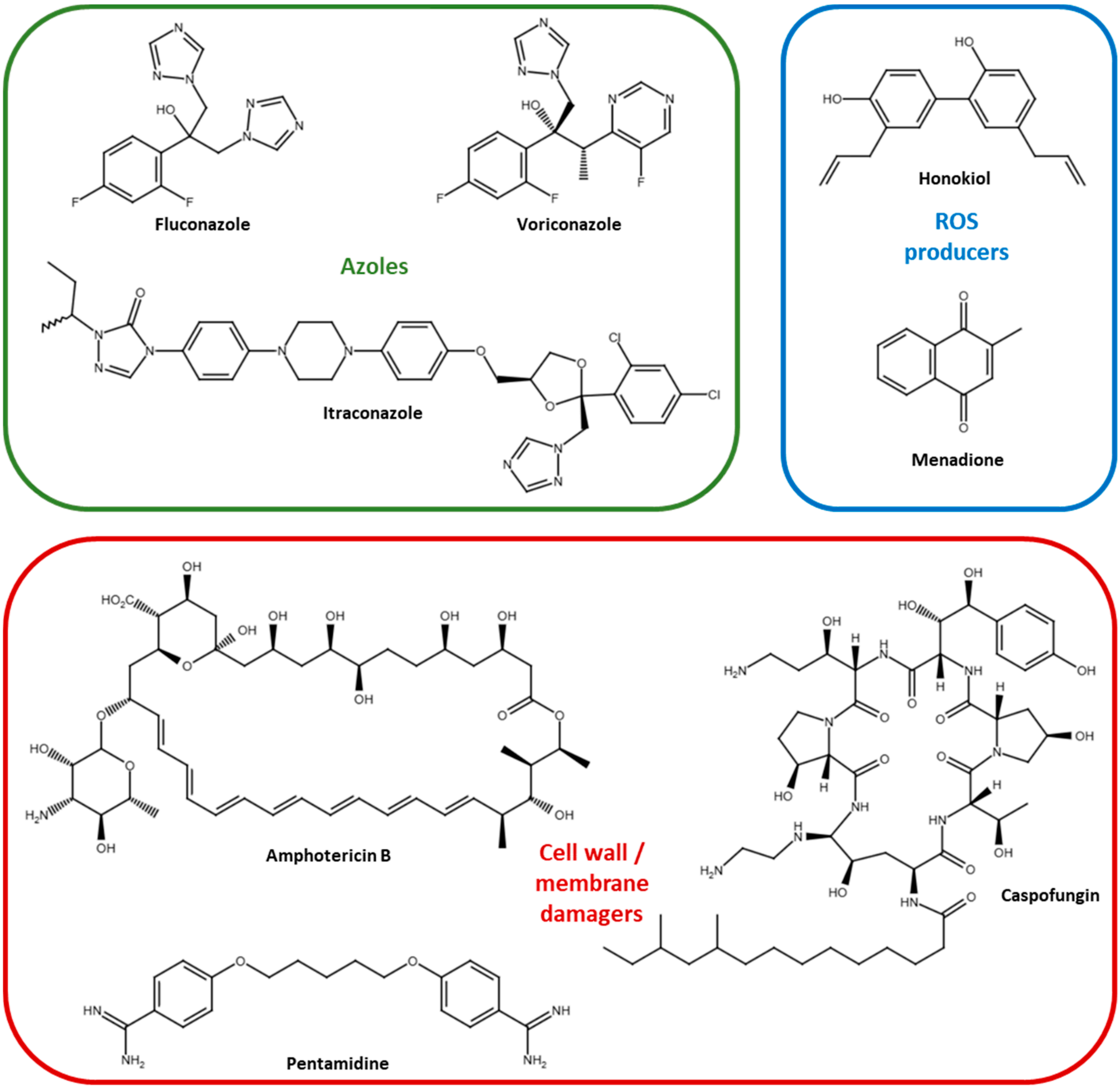

Figure S1. Structures of antifungal compounds used for combination therapies with auranofin.
